# Does digitalization boost companies’ outward foreign direct investment?

**DOI:** 10.3389/fpsyg.2022.1006890

**Published:** 2022-11-29

**Authors:** Cheng Peng, Shanshan Yang, Hui Jiang

**Affiliations:** ^1^School of International Finance and Trade, Sichuan International Studies University, Chongqing, China; ^2^Bartlett School of Environment, Energy and Resources, University College London, London, United Kingdom; ^3^School of Economics and Business Administration, Chongqing University, Chongqing, China

**Keywords:** digitalization, total factor productivity, financing constraints, OFDI, digital technology

## Abstract

The development of digital economy influences the digital transformation of companies while profoundly affecting the production efficiency, business model and overall strategy of firms, which has an important impact on business decisions, including foreign investment decisions. However, whether and how digitalization affects corporate OFDI has not been sufficiently empirically investigated. Taking Chinese listed companies in Shanghai and Shenzhen A-shares from 2009 to 2020 as samples, this paper constructs corporate digitalization indicators by using “text analysis method” and empirically tests the impact of digitalization on corporate OFDI and its path. The study finds that digitalization significantly promotes corporate OFDI. In terms of the influential mechanism, digitalization promotes corporate OFDI by improving total factor productivity and reducing financing constraints. In addition, external digital economy policies can provide favorable support for the digital transformation of firms. Meanwhile, the impact of digitalization on corporate OFDI is somewhat heterogeneous due to the different resource utilization efficiency and market environment. This study not only reveals the impact mechanism of digitalization on corporate OFDI, but also provides micro evidence for the deep integration of digital economy and real economy. Meanwhile, the findings have important implications for the formulation and implementation of digital policies.

## Introduction

The digital economy, as a new form of economy, is an important force in the implementation of major national strategies and plays an important role in promoting innovation-driven development strategies of a country. With the rapid development of the digital economy, it has had a huge impact on global investment. In the global investment report released by UNCTAD, it is stated that the digital economy can influence the scale and mode of investment of multinational enterprises by affecting their overseas operation mode, supply chain management mode, etc. Therefore, taking full advantage of the digital economic dividend and accelerating the flow of resource factors between countries is crucial to the healthy and rapid development of the global economy. With the drastic changes of international political environment and the outbreak of COVID-19 in these years, global supply chains and international economic exchanges have been seriously affected. Because the development of digital economy can improve the business efficiency of enterprises (Chu and Wang, 2022), improve the performance of products (Goldfarb and Tucker, 2019; Peng et al., 2022), facilitate people’s consumption (Hagiu, 2012; Vasenska et al., 2021; Song and Liu, 2022), and expand logistics channels (Sun et al., 2021), it is important for countries to adopt digitalization so as to minimize the negative impact of uncertainty and turbulence of international political and economic environment on international economic exchanges. As a developing country, China is much more vulnerable to the changes of international environment in the process of corporate going abroad. Consequently, there is a greater need for China to develop digital economy so as to facilitate OFDI.

Compared with developed countries, Chinese enterprises engaged in OFDI generally have problems of insufficient innovation capacity and relatively weak technological advantages in developing overseas markets. This restricts the improvement of international competitiveness of enterprises to a great extent, which is not conducive to the long-term development of enterprises. With the integration of digital economy and real economy, emerging technologies such as big data, 5G communication, blockchain and cloud computing have become important driving forces for innovation and development of firms, deep mining and flexible application of data have become key issues for corporate to gain new competitive advantages(Wu et al., 2021). In 2021, China’s Ministry of Commerce issued the Guidelines for Foreign Investment Cooperation in Digital Economy, which proposes that China should actively participate in the global industry chain of digital economy, optimize the “going global” strategic layout, use digital technology to promote digital transformation of firms, and build digital enterprises with global competitiveness. Therefore, it is imperative for Chinese firms to get involved in digital transformation, so as to improve the enterprises’ innovation ability, strengthen their global competitiveness, increase the efficiency of transnational investment, and thus promote their OFDI.

However, it is not very clear whether digital transformation will affect enterprises’ outbound investment and what the impact mechanism is. Most of literature mainly focused on the influence of digital infrastructure or development of digital economy in the host country on foreign investment of enterprises (Zhang, 2022), and believed that the digital infrastructure of the host country could effectively attract foreign investment (Zhou and Wu, 2021). Although this showed the influence of digital transformation on enterprises’ foreign investment to a certain extent, it did not explain how the development of digital economy affected their foreign investment from the perspective of enterprises themselves. Only a few literature focused on the topic of the influence of corporate digitalization on foreign direct investment, and preliminarily tested the relationship between them (Yang and Bi, 2019; Srinivasan and Eden, 2021; Stephenson et al., 2021). However, these researches contradict each other in the direction of impact of corporate digitalization on OFDI, and the influence mechanism also has not yet been revealed. Consequently, it is of great significance to further explore the impact of corporate digitalization on enterprises’ foreign direct investment behavior.

The paper takes Chinese listed companies in Shanghai and Shenzhen A-shares from 2009 to 2020 as samples to empirically analyze the relationship between corporate digitalization and OFDI, and examine the mediating roles played by total factor productivity and financing constraints. In addition, this paper analyzes the heterogeneous performance from the perspective of different ownership structure, industries and locations. The possible contribution of this paper are: (1) This paper examines the impact of corporate digitalization on enterprises’ foreign direct investment, which deepens the understanding of the consequences of corporate digital transformation and enriches the research on influencing factors of enterprise foreign investment behavior. (2) This paper examines the specific path of corporate digitalization affecting enterprises’ OFDI, so as to put forward meaningful suggestions for promotion of enterprises’ foreign direct investment.

This paper is structured as follows. In the next section, we present the theoretical analysis as well as the research hypotheses. We then describe the research design, including sample selection, data sources, variable measures, and model design, to empirically demonstrate the impact of corporate digitalization on OFDI. Finally, we discuss the empirical results and draw conclusions, and explore the potential implications of this study.

## Literature review

### Research on the economic consequences of enterprise digitalization

Many scholars have paid attention to the economic consequences of digitalization. Firstly, part of the research studies the economic consequences that digitalization can bring to the enterprise. Valaskova et al. (2021) empirically demonstrated that advanced computing, cutting-edge analytics, cyber-physical production systems, and artificial intelligence enable businesses to create additional value based on increased responsiveness and robustness, thus proving the value creation effect of digitalization. Hopkins and Siekelova (2021) argued that integrating internet of things-based decision systems in production processes help companies to automatically collect inspection data and optimize manufacturing operations processes. Different to them, Vial (2019) focused on the changes that digitalization can bring to enterprises from the perspective of total factor productivity, and realized that digital technology would play a positive contribution to the productivity of enterprises. Secondly, the other part of research studies the reduction of financing constraints that digital transformation can bring to enterprises from the perspective of the financial industry. According to Wu (2015), data science could present fascinating prospects for the new threats in the global financial system, and the “catfish effect” brought by digital finance can change the competitive situation of financial industry, and in the process of digital transformation, it would also have an impact on the technology and services of the financial industry, thus enhancing the efficiency of the financial system and creating a favorable environment for enterprise financing. Tong et al. (2020) and Wan et al. (2020) both concluded that digitalization has to some extent broken the original constraints of time and space, making financial services more inclusive and the emerging risks in financial system easy to be monitored (Morales et al., 2022), easing the financing constraints while also promoting enterprise technological innovation. Zhang and Wu (2020) believed that digital technology can strengthen the degree of information between borrowers and lenders and use an intelligent risk control system to reduce corporate financing constraints as well as default risks. Willi and Allan (2018) found that digital finance, based on online internetization, can break through the traditional physical distance and broaden financing channels, and also can effectively control the problem of financial managers using financial reports for profits in terms of anti-fraud and big data traceability, which would alleviate the problem of financial constraints faced by the firms.

### Research on the impact of enterprise characteristics on OFDI

In recent years, more and more scholars have gradually focused on the impact of differences in enterprises’ internal endowments, productivity, and financing capacity on their OFDI decisions. Gao and Zhang (2017) conducted an empirical study on Chinese service sector companies and found that productivity, capital, and labor all contribute significantly to corporate cross-border investment. Melitz (2003) argued that companies with low productivity focus mainly on their home market, and only companies with high productivity have the energy and ability to conduct overseas investment operations. Wang et al. (2020) found that in companies with higher capital intensity, overseas investment decisions are more likely to be influenced by productivity. In addition, in recent years, scholars have also focused on the role of financing constraints on corporate overseas investment. Qiu and Liu (2016) argued that enterprises with higher financing capacity are more willing to make OFDI. Wang (2009) emphasized that if without financing constraints, companies will over invest, while the financing constraint will play a restraining role and effectively curb the company’s over investment behavior, which also shows the negative impact of financing constraint on the company’s overseas investment (Dong and Chen, 2021).

### Research on the impact of digitalization on enterprise OFDI

In UNCTAD’s 2017 report on global investment, it was noted that the digital economy can have an impact on the way enterprises expand abroad, the scale and direction of cross-border investment, etc. The digital economy can reconfigure international production, create new market entry channels, thus exert influence on enterprise OFDI. From a macro perspective, Backer and Flaig (2017) argued that the development of the digital economy can enhance a country’s position in the division of labor in the global value chain. Multinational enterprises will not only consider the technological development level of the host country, but also tend to invest in countries with better digital infrastructure when choosing transnational investment destinations. Fan (2020) found from the two-way perspective of the host country and home country that the application of digital technologies such as the Internet can promote bilateral investment of a country by reducing costs. From a micro perspective, Stephenson et al. (2021) argued that global investment has been affected by the spread of the epidemic, but the resources provided by digital technology can accelerate the transformation of production and thus promote the sustainable development of enterprise OFDI. Yang and Bi (2019) found that because “internet+” based big data analysis can effectively reduce the risk caused by mismatch of proprietary assets of the firm, thus promoting foreign direct investment of enterprises aiming at obtaining proprietary assets from abroad. Dai and Han (2021) argued that Internet development can promote enterprise OFDI by promoting exports and alleviating financing constraints. However, Banalieva and Dhanaraj (2019) found that digitalization would also reduce enterprises’ OFDI as the development of digital technology enables them to participate in the international market without real investment in foreign countries (Qi and Chai, 2020).

## Theoretical analysis and research hypothesis

### Digitalization and enterprise OFDI

As all know, it is important for enterprises to obtain timely and effective information for OFDI. However, Li et al. (2020) found that although research institutions have invested heavily in overseas-related investment research, the overall quality of research output is not high, which is difficult to meet the needs of national policy making and enterprises investment decisions. Although some studies do help enterprises grasp the characteristics of overseas consumer markets to implement overseas investment strategies, there are still problems of fragmentation and lack of transparency. Therefore, the difficulty in obtaining information is a major problem that hinders enterprises from investing overseas. With the rapid development of the digital economy, most enterprises can use big data, cloud computing, blockchain and other technology for reform and development, improving the information collection capability. When enterprises use the information advantage to obtain the demand information of overseas consumers in a timely manner, they will be able to adjust their product strategies and production decisions more quickly, which will play a positive role in enterprises’ overseas investment (Chen and Zhou, 2018; Huang et al., 2020). In addition, Huang et al. (2019) argued that the deep integration of traditional enterprises with digital transformation can help enterprises to carry out intelligent production. It can improve the production efficiency of enterprises, promote the innovative development of industrial profitability models and enhance the international competitiveness of enterprises. At the same time, Xie et al. (2020) holds that data can deeply integrated with labor, knowledge, technology, management and capital, which are factors of production, forming more realistic productivity factors to help enterprises improve competitiveness. Wade and Vochozka (2021) argued that sustainable manufacturing Internet of Things can facilitate network collaboration and help companies gain advantages of scale. Enterprises can take advantages of their unique competitive advantages to actively participate in the international market division of labor.

In addition, according to the theory of market internalization, incomplete market will lead to the rise of corporate transaction costs. Digitalization can help enterprises optimize their value network, internalize part of the external market. The use of digital technology enables corporate to bypass intermediaries and communicate efficiently with business partner terminals. Shi and Li (2020) argued that the use of such information technology can not only reduce the communication costs of firms, but also help enterprises optimize their organizational structure, improve resource utilization efficiency and reduce enterprise management costs, improving the operational efficiency of enterprises to prepare for participation in the international market division of labor. Besides, Shi (2016) concluded that technological advances such as the Internet will reduce the demand for labor, the production cost of products and the cost of overseas operations in the process of enterprise OFDI, which is conducive to the further expansion of overseas markets.

**Figure 1:**
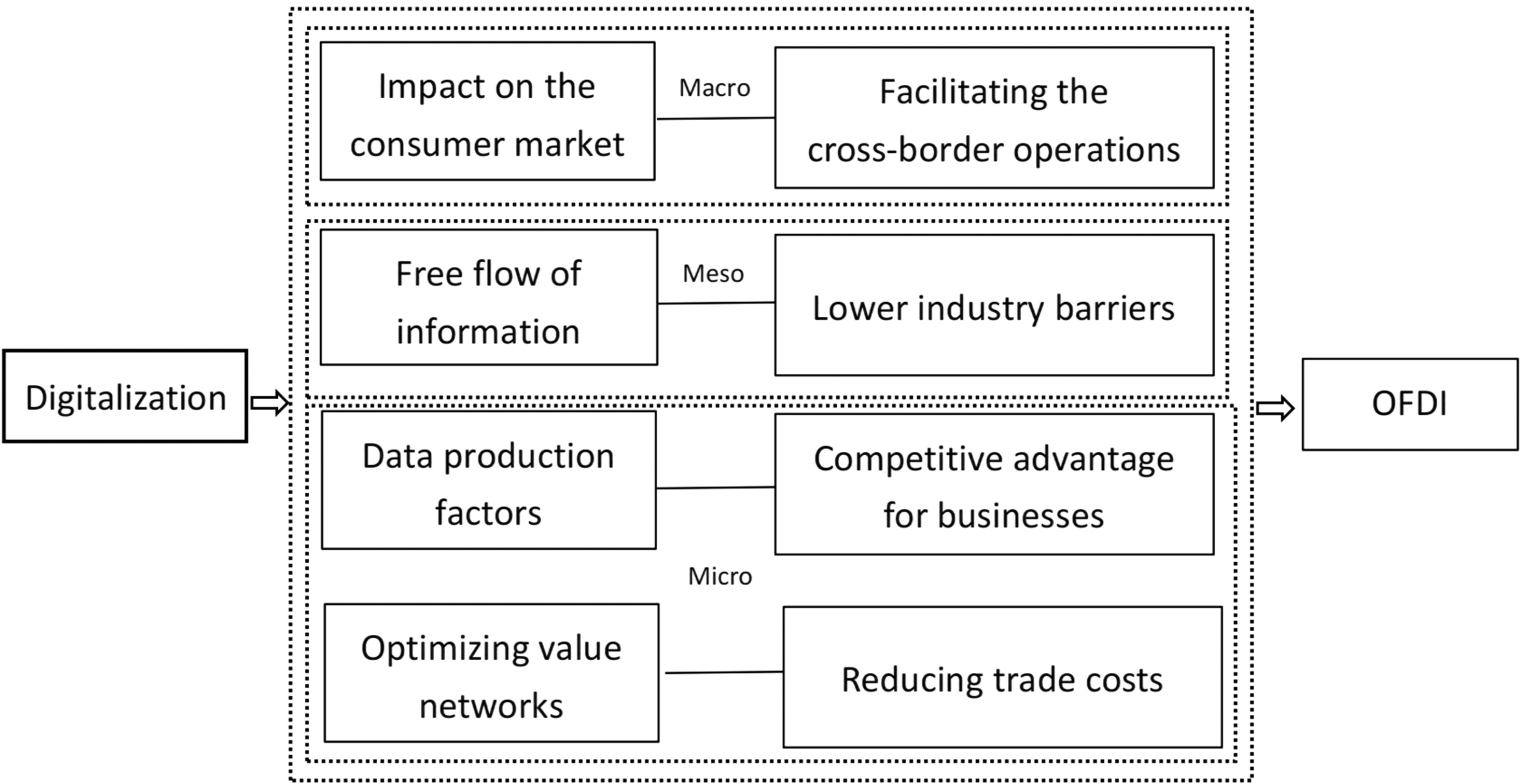
Analysis of the mechanism of digitalization on enterprise OFDI.

Therefore, based on the above analysis, this paper proposes research Hypothesis 1. (The simplified analysis logic is shown in Figure 1).

*H1*: Digitalization can significantly promote enterprise OFDI.

### Digitalization, total factor productivity and enterprise OFDI

Digitalization facilitates technological progress, efficiency improvement and cost reduction of enterprises, thus promoting the total factor productivity of enterprises. (1) In terms of technological progress, Chen et al. (2019) argued that the construction of digital infrastructure such as the Internet can significantly enhance innovation activities, which can improve the total factor productivity of enterprises. On the one hand, the internal technicians of enterprises can make use of the complete facilities and advanced technology to improve the efficiency of R&D, thus in turn effectively affecting corporate productivity (Bilan et al., 2020). On the other hand, digitalization provides a new communication platform for both supply and demand sides of the market. Guo and Luo (2016) argued that corporate can use information from various platforms to quickly and accurately grasp market demand and mobilize corporate resources for innovation activities. The diverse needs of consumers become a powerful driver of corporate innovation, which promotes the technological progress and the total factor productivity of firms. (2) In terms of efficiency improvement, firstly, the digital development of enterprises is conducive to improving the efficiency of resource allocation. Digital platform construction facilitates the matching of supply and demand in the market, which promoting the improvement of resource allocation efficiency of the enterprise. Li et al. (2017) argued that a convenient information exchange platform can improve the effective allocation of labor resources by increasing the information symmetry in the labor market. This can alleviate the employment pressure while realizing the accurate matching of talents and jobs. Secondly, digital technology is conducive to improving the efficiency of resource integration. Due to the problems of transaction costs and information asymmetry in the real economy, the externalities of transactions cannot be solved. However, on the one hand, digital trading platforms can internalize the externality problem through price adjustment and improve the matching efficiency of the whole transaction. On the other hand, Sun et al. (2017) argued that the use of technology such as the Internet can reduce information asymmetry and increase the degree of information disclosure of enterprises on product prices and quality. This can create an open and transparent trading environment and credit system, promoting fair competition in the market and enhancing the efficiency of integration of resources across the society. Thirdly, Xiao (2018) studied that enterprise digitalization is beneficial to improve the communication efficiency of enterprises. On the one hand, digitalization is conducive to improving the internal communication efficiency of enterprises. The application of digital technology helps to achieve rapid exchange of information between enterprise departments at low cost and improve the efficiency of cooperation between departments. This leads to a shift from single linear innovation to parallel innovation of products, which can faster the speed of knowledge progress. On the other hand, digitalization facilitates the efficiency of communication between enterprises and consumers. The popularity of intelligent products using digital technology in life can quickly collect the feedback of user data. The analysis and integration of big data helps enterprises to find out where to improve their products, which in turn improves the efficiency of their knowledge progress. (3) In terms of cost reduction, the digitalization of enterprises is conducive to the reduction of information transmission costs, transaction costs and production costs of firms. The information obtained through big data and other platforms is more accurate and reliable, which can reduce the cost of information transfer within as well as outside the company. Han et al. (2014) pointed out that the use of information technology can enhance the degree of corporate information disclosure in the process of conducting transactions. Both sides of the transaction have more symmetrical information, which helps to reduce transaction costs and alleviate market imbalances. In addition, as mentioned earlier, digitalization of the enterprise helps to enhance the R&D investment of the enterprise. This increase in innovation capacity can significantly contribute to the growth of corporate total factor productivity by reducing the production costs and their dependence on labor and capital.

The increase in total factor productivity also helps to promote enterprise OFDI. Most of the current studies on total factor productivity and OFDI are based on Melitz’s (2003) theory of heterogeneous enterprise trade, in which he introduced enterprises productivity differences into the model for the first time to explain corporate foreign trade and investment behavior. In the study of Helpman et al. (2014), he introduced the heterogeneity theory based on the “proximity-concentration” hypothesis and found that enterprises choose different business practices according to their productivity by constructing the HYM model. Enterprises with higher productivity will participate in international markets by investing abroad, while enterprises with lower productivity will choose to export to other countries. A larger number of scholars have subsequently tested the HYM model and indeed reached similar conclusions. Firstly, scholars Gao and Zhang (2017) found through their study that enterprises with higher total factor productivity tend to have ownership advantages and can produce standardized products to achieve economies of scale with lower unit product costs. Such enterprises therefore rely on price competition to participate in international markets to expand their overseas market share. Secondly, industries with higher productivity are able to accumulate international business management experience through exports as a matter of priority. They can learn from the specifics of foreign markets and prepare for the location choice and other problems faced by OFDI later. Location advantage is one of the most important reasons for the success of foreign investment. In addition, exporting can change the consumption preference of the host country through demonstration effect, which may gain the support of the host country consumers and the success rate of enterprise OFDI will also be higher. Thirdly, when an industry has advanced management experience and high productivity, it necessarily requires its upstream and downstream industries to have the matching experience and degree of development in order to provide perfect integrated services. When this industry decides to enter the foreign market, it will also promote international investment in related industries through the ripple effect.

**Figure 2:**
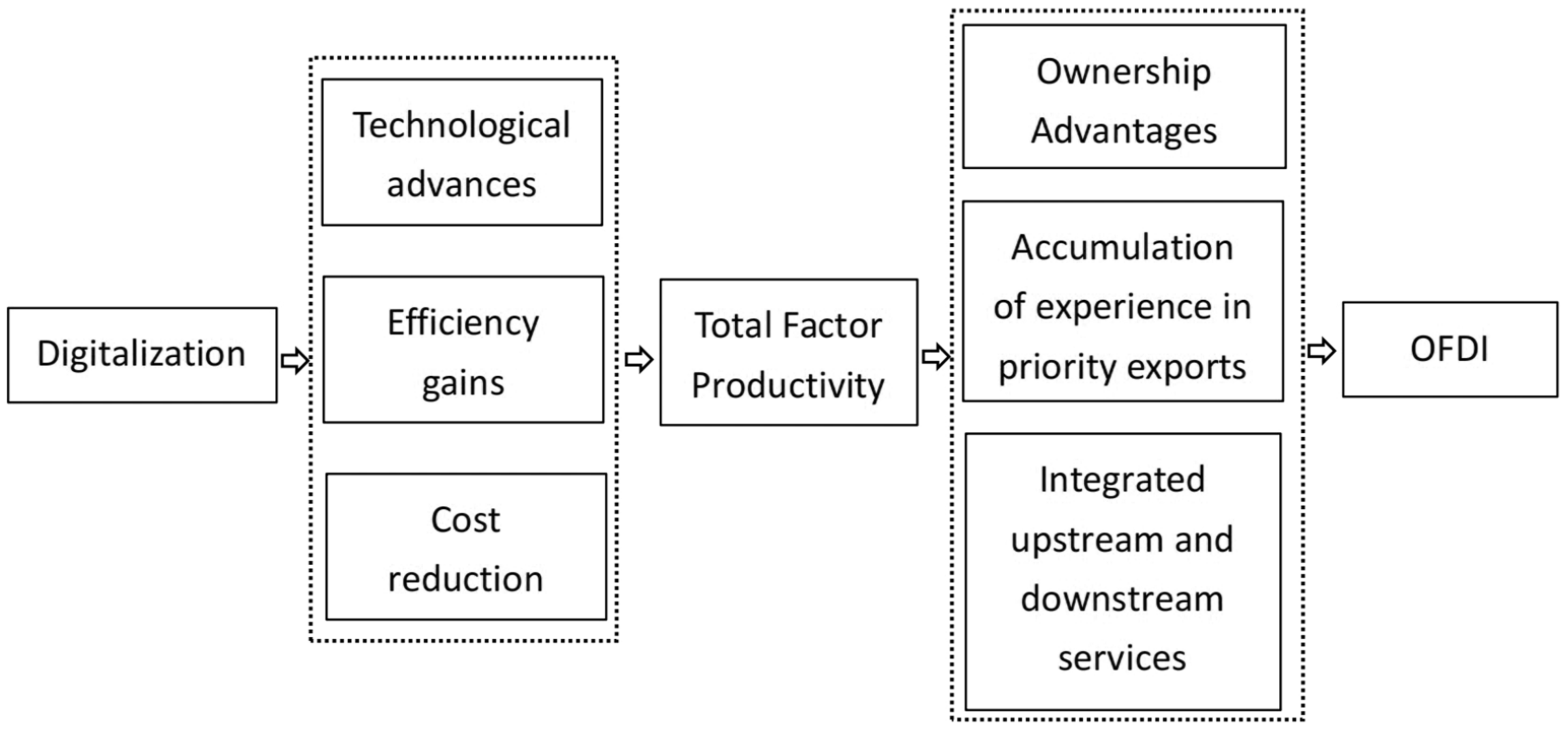
The mediating role of total factor productivity.

Therefore, based on the above analysis this paper proposes research Hypothesis 2 (The simplified analysis logic is shown in Figure 2).

*H2*: Digitalization can promote enterprise OFDI by enhancing total factor productivity.

### Digitalization, financing constraints and enterprise OFDI

The digitalization of enterprises can enhance their overall strength and improve the level of information disclosure through digital technology, which can reduce the financing constraints of enterprises. On the one hand, the use of digital technology in production can significantly improve the “hard power” of enterprises in terms of productivity, innovation output and product quality. It improves the business strength in all aspects and thus increases the risk resistance of the enterprises. Lewandowska et al. (2021) emphasized that financial support for investments is a very important lever for developing firms innovativeness. As analyzed above digitalization can significantly increase business productivity. Digital technology can help enterprises to specialize in the division of labor to improve product quality. This allows enterprises to devote more energy to innovation activities and to the production of their core products. In addition, the use of digital technology can facilitate internal organizational change and enhance the “soft power” of the enterprises. Digital technology enables low-cost communication and close cooperation between departments, which will foster a culture of harmony and unity within the enterprises and attracting more integrated talent to the enterprises. Banks and other investors often place great importance on the business conditions and future growth capabilities of enterprises when choosing investment objectives. Therefore, the improvement of the comprehensive ability of enterprises is conducive to solving the problem of difficult financing for enterprises. On the other hand, the information asymmetry between enterprises and investors may also lead to an increase in financing costs. Myers and Nicholas (1984) argued that due to information asymmetry, the suppliers of funds are unable to grasp the internal information of enterprises and tend to choose to increase the capital premium, which in effect increases the financing costs of enterprises. At the same time, due to the lack of full certainty of recovering the funds, banks will also appropriately reduce the loan amount or even unwilling to provide loans. However, with the widespread use of digital technology in traditional finance, digital technology such as Internet-based technology can effectively span the geographical distance limit and broaden financing channels. Willi and Allan (2018) argued that the use of digital finance in areas such as big data traceability and anti-fraud can effectively reduce managers’ whitewashing behavior of corporate statements, which can enhance the transparency of corporate information. In addition, the emergence and development of technology such as big data and cloud computing have also affected the auditing activities of enterprises. According to Qin (2014), big data focuses on data collection, mining and analysis, while cloud technology focuses on “computing” to provide corresponding solutions. All these techniques lead to more timely and transparent corporate disclosures by optimizing the use of audit results, the treatment of relevant relationship evidence and the development of audit models. Ionescu (2004) also argued that the use of fintech inherently enhances the ability of private participants to coordinate commercially viable financial claims. Therefore, disclosure of relevant information will help investors make better decisions. The improvement of corporate information transparency is conducive to reducing the financing constraints of enterprises.

The reduction of financing constraints can also promote enterprise OFDI. Scholars have only slowly incorporated financial factors into the study of corporate behavior with the rise of incomplete markets and adverse selection theory in the 1970s. Since investors do not have all the information about the corporate, there will be a risk premium in providing funds. This shows that enterprises will inevitably incur certain costs in the process of financing, resulting in financing constraints. Earlier scholars mostly linked financing with exporting. Muuls (2008) studied heterogeneous enterprise trade from the perspective of corporate internal and external financing channels and found that enterprises with financing constraints would abandon exporting because they could not pay the fixed costs required to export. Therefore, enterprises with lower financing constraints are more likely to export. Buch et al. (2010) found that OFDI requires higher fixed costs and is subject to higher financing constraints than exporting. Even if a enterprise has a large domestic market share, if it cannot obtain financial support through external financing, it is unable to pay the high cost of building a factory overseas. Specifically, financing constraints can affect enterprises OFDI in three ways: by affecting financing costs, innovation inputs and resource allocation. In terms of financing costs, too high financing costs will increase the financial burden of enterprises and prevent them from investing funds to optimize their organizational structure, which is not conducive to their long-term development and will also increase their international investment risks. In addition, Wang et al. (2015) argued that some enterprises cannot afford the excessive financing costs, so they will appropriately adjust the scope of overseas investment, which may lose some favorable investment opportunities in the long run. In terms of innovation investment, since enterprise R&D projects require huge sum of funds, which can not be borne by the internal financing market of corporate. Therefore, external financing is an important source of funding for enterprise innovation. However, corporate innovation activities are also characterized by uncertainty of returns, so investors often choose to hedge their bets when faced with risk. Hall (2002) argued that innovation is an important driver of internationalization, helping enterprises to improve their international competitiveness and gain a leading position in the international market. However, such a “financing gap” hinders the R&D activities of enterprises and prevents them from taking the lead in international competition, which may slow down the process of foreign investment of enterprises. In terms of resource allocation, the greater the financing constraint means that it is more difficult for enterprises to obtain funds from external sources, so they will turn to use their own funds. In this situation, the enterprise has to change the way they use funds. This would have a negative impact on the overall resource allocation, which is not conducive to business production and would affect outbound investment decisions.

**Figure 3:**
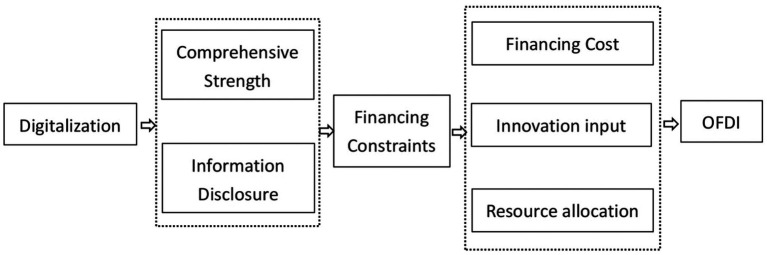
The mediating role of financing constraints.

Therefore, based on the above analysis, this paper proposes research hypothesis 3 (The simplified analysis logic is shown in Figure 3).

*H3*: Digitalization can promote enterprise OFDI by reducing financing constraints.

## Materials and methods

### Data and sample

This paper selects the listed enterprises in Shanghai and Shenzhen A-shares from 2009 to 2020 as a sample to study the impact of digitalization on enterprise OFDI. The reason why the sample period was selected from 2009 is, on the one hand, that online shopping activities represented by the “Double 11” have been popular in China since 2009. The digital economy has begun to profoundly affect people’s behavior and habits, and has also played an important role in the adjustment of business strategies of enterprises. On the other hand, in 2009, the world economy began to slowly recover from the haze of the financial crisis, Chinese enterprises’ foreign direct investment has also entered a stage of steady development. Since some data of listed companies in China can only be obtained until 2020, the sample period ends in 2020. The data selected in this paper are obtained from Wind database and CSMAR database. On this basis, the selected data are processed as follows: Firstly, this paper focuses on the impact of digitalization of real enterprises, so financial enterprises are excluded. Secondly, the data of enterprises with special characteristics of ST and *ST category and the sample of enterprises with serious deficiencies are excluded. At last, we get a sample including 20,627 observation data of 3,181 listed companies in total. Among them, 7,276 data are about the enterprises which participate in OFDI during the sample year. At the same time, the sample enterprises are distributed in 17 industries. Of the 3,181 sample enterprises, 972 are state-owned enterprises and 2,209 are non-state-owned enterprises; There are 2,285 enterprises in eastern China, 475 enterprises in western China and 421 enterprises in central China.

### Variable selection

#### Explanatory variables

Enterprise digitalization (DIG). The current measurement of digitalization is mostly focused on the measurement of digital economy of regions or industries at macro level. The measurement of digitalization indicators at the micro level is still very challenging. In the few studies on enterprise digitalization, scholars mainly use information assets, technicians and communication infrastructure to measure enterprise digitalization, but none of these indicators can well reflect the true degree of enterprise digitalization For example, Li et al. (2015) use the percentage of intangible assets related to digitalization to measure the density of enterprise informatization. Although these indicators can reflect the degree of enterprise digitalization to some extent, the indicator is susceptible to the influence of corporate fake investments. Other scholars, such as Wang et al. (2017) used the form of questionnaires to calculate the proportion of enterprise technicians, the application of information infrastructure and other data as the proxies of digitalization. However, this method is not very representative because of the limited sample size and subjectivity. In addition, Yang and Liu (2018) used text analysis to capture terms related to information technology such as the Internet in enterprises to measure digitalization, but this indicator only focuses on the use of Internet technology, which cannot fully reflect the degree of digitalization of enterprises. Stankovic et al. (2021) used a two-stage procedure to integrate CRITIC and TOPSIS as a weighting and aggregation method to assess the digital competitiveness of European countries. However, this approach focuses on the measurement of digital competitiveness at the national level rather than at the enterprise level.

The current development of big data and computing technology can provide more quantitative statistical methods for the measurement of digitalization. Nowadays, more and more scholars begin to use “text analysis” to identify the frequency of keywords. If an enterprise uses keywords more frequently in its annual report, the more importance it attaches to the keywords. It can also reflect the future policy orientation and development priorities of enterprises.

Therefore, referring to Wu et al. (2021), this paper quantifies the digitalization of enterprises by using the “text analysis” to capture the key words about “digitalization” in the annual reports of enterprises from six dimensions and to conduct word frequency statistics. The specific methods are as follows: firstly, collate the annual reports of listed companies from 2009 to 2020. In this paper, the annual reports of all A-share listed companies in Shanghai and Shenzhen exchanges are collected by Python crawler function, and all text contents are extracted by Java PDFbox library, which is used as a data pool for the subsequent screening of “digitalization” feature words. Secondly, construct the “enterprise digitalization” keyword thesaurus. Based on the definition of digitalization and the literature on the topic of enterprise digitalization (Wu et al., 2021), this paper summarizes and sorts out the specific keywords related to enterprise digitalization, mainly including “big data,” “cloud computing,” “blockchain,” “artificial intelligence,” “Internet” and “digital information technology.” The specific terms are shown in Table 1. Thirdly, count the frequency of the key words of “enterprise digitalization” in the annual reports of listed companies. Based on the data pool formed by the text extraction of listed companies’ annual reports, the python crawler function is used to search, match and count the frequency of the feature words in Table 1. The total number of word frequencies is then summed up to build indicator of enterprise digitalization. Considering the “right bias” feature of such data, this paper takes the logarithm of these numbers.

**Table 1:** Construction of “enterprise digitalization” thesaurus.

Category	Keywords
Digital Information Technology	digitalization, digital marketing, digital technology, digital technology, digital operation, digital terminal, digital economy, digital trade, digital system, digital supply chain, digital finance, unmanned retail, information age, information technology, information integration, information communication, etc.
Artificial Intelligence	smart era, smart construction, smart business, intelligence, biometrics, face technology, voice recognition, autonomous driving, identity verification, 3D printing, 3D technology, 3D tools, AI, robotics, machine learning, 5G, etc.
Cloud Computing	cloud computing, cloud services, cloud, Internet of Things, graph computing, stream computing, etc.
Blockchain	blockchain, digital currency, smart financial contracts, etc.
Big Data	big data, data integration, data fusion, data information, data management, data assets, data mining, data visualization, etc.
Internet	Internet, electronic payment, mobile payment, e-commerce, cross-border e-commerce, e-commerce platform, electronic technology, electronic technology, online, online and offline, O2O, B2B, C2C, P2P, C2B, B2C, etc.

#### Explained variables

Enterprise outward foreign direct investment (OFDI_1 and OFDI_2). The existing literature usually use the database of the Ministry of Commerce’s “Directory of Outward Investment Enterprises” to match the OFDI data. This directory is relatively old (only updated to 2015). Therefore, this paper relies on the database of overseas affiliates in CSMAR to measure the OFDI of enterprises in terms of both the willingness and the level of OFDI. OFDI_1 (willingness of OFDI) is defined as a value of 1 if the overseas affiliates of the enterprise have made OFDI in the year, otherwise defined as 0. OFDI_2 (level of OFDI) is a comprehensive indicator based on the entropy method, which is based on the volume of OFDI by overseas affiliates, the number of overseas subsidiaries and the number of overseas countries and regions entered by the enterprises. This method can calculate the weights of each indicator based on objective facts by using the entropy weighting method and then synthesize the final indicator score. It can well avoid the influence of subjective factors (Tang and Han, 2001). The composite indicator determined according to the entropy method is in the range of 0–1. To better observe the regression results, this paper defines the level of enterprises’ foreign direct investment by multiplying the final result by 100.

#### Mediating variables

Total factor productivity (TFP). It is used to describe the efficiency of the utilization of human, material and financial resources of enterprises. In this paper, the LP method is used to measure total factor productivity.

Financing constraint (FC). There are many methods to measure variable financing constraints. However, most of the methods rely on financial indicators that are prone to endogenous problems, rather than directly related to financing constraints. To avoid this deficiency, this paper adopts the financing constraint variable (SA indicator) designed by Hadlock and Pierce (2010). It is constructed using only two variables, the enterprise size and age, which do not vary much over time and are highly exogenous. The specific formula is SA = −0.737*Size+0.043*Size^2–0.040*Age. Size is the natural logarithm of the enterprise size and Age is the length of time that the enterprise has been established. According to Lu and Zhang (2014), the SA indicator is negative and the larger the absolute value of SA indicator, the lower the degree of financing constraint faced by the enterprise.

#### Control variables

The percentage of enterprise fixed assets (PPE) is measured by the proportion of fixed assets to total assets in the current year. If an enterprise invests more in fixed assets, the more powerful it is to compete in the international market. The scale of enterprise liabilities (LEV) is measured by the ratio of total liabilities to total assets of the enterprise in the current year. The size of liabilities affects the financing cost of enterprises, which in turn has an impact on foreign investment. Capital intensity (K) is measured by the ratio of fixed assets to the number of employees of the enterprise in the current year, referring to the approach of Wang et al. (2015). Theoretically, China has a comparative advantage in labor-intensive industries, while capital intensity may have a different impact on outward investment. Outward investment is often costly, so the relationship between the capital intensity and OFDI is uncertain and it is necessary to add capital intensity as a control variable. Profitability (ROA) is measured by the ratio of total operating income to total assets of the enterprise in the current year. Profitability is an important motivation for enterprises to seek overseas markets. As the domestic market tends to be saturated or the competition is too fierce, some enterprises turn their attention to international markets for survival and growth when their capital allows. Export (EXPORT), referring to Liu et al. (2015), according to the financial statements of listed enterprises, if a enterprise does not make foreign investment but has overseas operating income, it is considered to have exported. In this situation, defining the export indicator as 1, otherwise it is 0. Growth of the enterprise (GROW) is measured by the growth of enterprise revenue, which can reflect the development trend and future potential of the enterprise. Tobin’s Q value is measured by the ratio of market value to total assets at the end of the period. A higher ratio indicates a higher return on business investment of the enterprise. Net working capital (NWC) is measured by the ratio of the change in net working capital to total assets between the current year and the previous year. It reflects the liquidity of the enterprise. A reasonable size of NWC is essential for the enterprise OFDI. All variables involved in this paper are defined as shown in Table 2.

**Table 2:** Definition of all variables.

Variables	Variable definition
Willingness of OFDI (OFDI_1)	1 for enterprise OFDI made in the current year, 0 otherwise
Level of OFDI (OFDI_2)	Total volume of OFDI by enterprises
	The number of overseas subsidiaries of enterprises
	The number of overseas countries and regions entered by the enterprises
Digitalization (DIG)	Statistics of related keywords in annual reports of listed enterprises
Total factor productivity (TFP)	LP method
Financing constraints (FC)	SA Indicator
Percentage of fixed assets (PPE)	Fixed assets in year *t*/total assets in year *t*
The scale of liabilities (LEV)	Total liabilities at the end of year *t*/total assets in year *t*
Capital intensity (K)	Net fixed assets/total number of employees
Profitability (ROA)	Total operating income at the end of year *t*/total assets in year *t*
Export (EXPORT)	Current year exports take 1, otherwise take 0
Growth of the enterprise (GROW)	[Total operating income in year *t*–(*t*–1) total operating income in year *t*]/(*t*–1) total operating income in year *t*
Tobin’s *Q* value (*Q*)	Market value/total assets at end of period
Net working capital (NWC)	[Net working capital in year *t*–(*t*–1) net working capital in year *t*]/total assets in year *t*

### Model setting

The model 1 is first developed to examine the direct influence of digitalization on the enterprise OFDI.


$$\mathrm{Model}\;1:{\mathrm{OFDI}}_{ft}=\alpha_{0}+\alpha_{1}{\mathrm{DIG}}_{ft}+\alpha_{j}X_{ft}+\gamma_{i}+\delta_{t}+\varepsilon_{ft}.$$

OFDI_ft_ in model 1 denotes denotes the OFDI of the enterprise *f* in year *t*, including OFDI_1 (willingness of OFDI) and OFDI_2 (level of OFDI); DIG*_ft_* denotes the degree of digitalization of the enterprise *f* in year *t*; *X* denotes a set of control variables; *γ_i_* denotes fixed effect of industry; *δ_t_* denotes fixed effect of time; ε denotes a random error term. Since OFDI_1 is a dummy variable, this paper will use logit regression for the model on OFDI_1. In addition, all models on OFDI_2 in this paper are tested by fixed-effects models. After Hausman test, the final value of *p* is 0.0000 < 0.05, which rejects the original hypothesis. Therefore, it is feasible to choose fixed-effects model to test models on OFDI_2, where both time and industry are controlled.

In addition, this paper will explore the possible mechanisms between digitalization and enterprise OFDI. Based on the theoretical analysis in this paper, it is tested whether the total factor production and financing constraints are mediating variables between digitalization and enterprise OFDI. The specific steps are as follows: after test the significance of coefficient *α*_1_ in the model (1), the linear regression equations of DIG on the mediating variables *W* (including TFP and FC) are then constructed separately. Finally the linear regression equations of DIG and mediating variables *W* (including TFP and FC) on OFDI are constructed. According to the process of testing mediating effects proposed by Wen and Ye (2014), the coefficients of the variables in each equation are to be tested in turn. On the baiss of coefficient *α*_1_ passing the significance test, if *λ*_1_ is not significant, but both *β*_1_ and *λ*_2_ are significant, there is a complete mediation effect; if *λ*_1_, *β*_1_and *λ*_2_ are all significant, there is a partial mediation effect when *β*_1_*λ*_2_ have the same sign with *λ*_1_, and there is a masking effect when *β*_1_*λ*_2_ and *λ*_1_ have different signs. The mediation effect models are shown as follows:


$$\mathrm{Model}\;2:\;\;W_{ft}=\beta_{0}+\beta_{1}{\mathrm{DIG}}_{ft}+\beta_{j}X_{ft}+\gamma_{i}+\delta_{t}+\varepsilon_{ft}.$$

$$\mathrm{Model}\;\;3:{\mathrm{OFDI}}_{ft}=\lambda_{0}+\lambda_{1}{\mathrm{DIG}}_{ft}+\lambda_{2}W_{ft}+\lambda_{j}X_{ft}+\gamma_{i}+\delta_{t}+\varepsilon_{ft}.$$

## Results

### Descriptive analysis

Table 3 presents the descriptive statistics for all variables in the regressions. All continuous variables involved in this paper are winsorized at the 1st and 99th percentiles to mitigate the influence of outliers. In Table 3, group (1) is the result of descriptive analysis including OFDI_1, where the sample size of all variables is 20,627. Group (2) is the result of descriptive analysis including OFDI_2. The sample size of all variables is 7,276, among which the OFDI_2 samples are all those who have made OFDI. According to the above descriptive statistics, the digitalization of enterprises (DIG) presents a large standard error, indicating that different enterprises attach different importance to digitalization, which shows the same results of the study of Wu et al. (2021). The mean value of OFDI (OFDI_2) is 1.360. The maximum value is 11.29, while the minimum value is 0.249. Overall, the level of Chinese enterprises OFDI is low. There is still a large gap between the investment levels of different companies. In terms of control variables, indicators such as enterprise growth (GROW) and profitability (ROA) are characterized by low average values, indicating that most enterprises are developing slowly. Only a relatively small number of enterprises have achieved rapid growth in the last decade.

**Table 3:** Results of descriptive statistics of main variables.

Variables name	Observations		Average value		Standard deviation		Minimum value		Maximum value	
	(1)	(2)	(1)	(2)	(1)	(2)	(1)	(2)	(1)	(2)
OFDI_1 and OFDI_2	20,627	7,626	0.384	1.360	0.486	1.453	0	0.249	1	11.29
DIG	20,627	7,626	3.207	3.584	1.317	1.251	0.693	0.693	6.312	6.384
TFP	20,627	7,626	9.072	9.323	0.972	1.000	6.872	7.240	12.09	12.27
FC	20,627	7,626	−3.808	−3.778	0.220	0.230	−4.476	−4.430	−3.163	−3.060
PPE	20,627	7,626	0.219	0.200	1.099	1.120	0.002	5.357	0.693	11.45
LEV	20,627	7,626	0.421	0.423	0.147	0.130	0.055	0.056	0.884	0.859
K	20,627	7,626	0.452	0.429	0.557	0.513	0.013	0.003	5.335	0.641
ROA	20,627	7,626	0.625	0.634	0.370	0.344	0.084	0.102	2.453	2.350
EXPORT	20,627	7,626	0.627	0.832	0.484	0.374	0	0	1	1
GROW	20,627	7,626	0.155	0.156	0.305	0.267	−0.540	−0.463	2.414	1.645
Q	20,627	7,626	1.978	1.952	1.028	1.040	0.881	0.858	7.295	7.780
NWC	20,627	7,626	0.017	0.017	0.077	0.075	−0.246	−0.221	0.282	0.328

### Baseline regression results

Table 4 reports the regression results of the impact of digitalization on the willingness of enterprise OFDI (OFDI_1). In column (1) and column (2), it can be seen that the estimated coefficient of digitalization (DIG) is significantly positive, indicating that digitalization can promote the willingness of the enterprise to invest abroad. The higher digitalization indicates a greater willingness of enterprises to expand in overseas markets. With the inclusion of control variables, the results in column (2) shows that the coefficient of digitalization (DIG) is still significantly positive. The variance inflation factor (VIF) of this model are less than 3.6 and the mean value is 2.10, indicating that there is no problem of multicollinearity. Therefore, the hypothesis 1 has been proved. The control variable capital intensity (K) is significant at the level of 1%, which means the higher the capital intensity, the greater the capital advantage of enterprises for overseas investment. For profitability (ROA), there is a significant positive relationship with OFDI, indicating that profitability is an important motivation for enterprises to seek overseas markets. Whether a enterprise exports or not (EXPORT) is also closely related to OFDI, which is significant at the 1% level, indicating that export enterprises usually make overseas investment at the same time. In addition, for the robustness of regression results, this paper replaces DIG with DIG_t-1_ and DIG_t-2_, and lists the results in column (3) and (4) respectively. At the same time, it changes the sample period to 2012–2018, and lists the results in column (5) and (6) respectively. The table reveals that there is no significant change in results of these columns, showing the regression results of model (1) are robust and reliable.

**Table 4:** OFDI_1 basic regression results.

Variables	OFDI_1					
	(1)	(2)	(3)	(4)	(5)	(6)
DIG	0.241*** (19.505)	0.239*** (16.902)	0.236*** (14.787)	0.234*** (13.628)	0.232*** (15.565)	0.241*** (13.991)
PPE		−1.396*** (−9.708)	−1.483*** (−9.079)	−1.548*** (−8.798)		−1.667*** (−9.402)
LEV		0.712*** (7.688)	0.758*** (7.093)	0.648*** (5.517)		0.814*** (7.126)
K		0.283*** (7.665)	0.334*** (7.924)	0.343*** (7.622)		0.384*** (8.188)
ROA		0.228*** (4.906)	0.239*** (4.524)	0.255*** (4.472)		0.273*** (4.780)
EXPORT		1.585*** (43.518)	1.608*** (39.566)	1.617*** (37.091)		1.582*** (35.327)
GROW		0.016 (0.287)	0.084 (1.343)	0.067 (0.966)		0.039 (0.599)
Q		−0.034* (−1.899)	−0.019 (−0.937)	−0.037* (−1.725)		−0.064*** (−2.815)
NWC		−0.078 (−0.364)	0.044 (0.176)	0.084 (0.313)		0.004 (0.014)
CONS	−2.691*** (−23.914)	−3.968*** (−27.058)	−4.453*** (−24.820)	−3.828*** (−23.988)	−1.379*** (−19.862)	−2.679*** (−22.127)
Year	Control	Control	Control	Control	Control	Control
Industry	Control	Control	Control	Control	Control	Control
N	20,627	20,627	16,049	13,392	12,555	12,555
R^2^	0.068	0.152	0.145	0.132	0.023	0.115

*T*-statistic values are reported in parentheses in the table. ^***^, ^**^, and ^*^ indicates that the regression results passed significance tests at the 1, 5, and 10% confidence levels, respectively.

After verifying that corporate digitalization significantly contributes to the willingness of enterprises’ OFDI, Table 5 further reports the results of the impact of corporate digitalization on the level of enterprise OFDI (OFDI_2). In column (1) and column (2), it can be seen that the estimated coefficient of digitalization (DIG) is significantly positive at the 1% level. This indicates that corporate digitalization can significantly increase the level of enterprise OFDI. For those companies invest abroad, the higher the degree of digitalization, the greater the amount of enterprise OFDI. Therefore, the hypothesis 1 has been further proved. The results in column (2) show that the coefficient of LEV is significantly positive, indicating that the more debt, the more foreign direct investment of enterprises. In addition, this paper still replaces DIG with DIG_t-1_ and DIG_t-2_ [the results are in column (3) and column (4) respectively], and change the sample period to 2012–2018 for further analysis [the results are in column (5) and column (6)], there is no significant change in results, showing the results of the regression are robust and reliable.

**Table 5:** OFDI_2 basic regression results.

Variables	OFDI_2					
	(1)	(2)	(3)	(4)	(5)	(6)
DIG	0.478*** (12.038)	0.128*** (5.964)	0.123*** (4.922)	0.130*** (4.628)	0.106*** (4.119)	0.101*** (3.976)
PPE		−0.420* (−1.831)	−0.004 (−0.014)	−0.132 (−0.390)		−0.620** (−2.222)
LEV		1.585*** (11.657)	1.550*** (8.966)	1.763*** (8.806)		1.638*** (9.611)
K		0.115* (1.956)	0.017 (0.238)	0.053 (0.597)		0.110 (1.555)
ROA		0.070 (0.951)	0.015 (0.165)	−0.041 (−0.366)		−0.042 (−0.440)
EXPORT		0.157** (2.256)	0.109 (1.278)	0.128 (1.270)		−0.063 (−0.749)
GROW		−0.032 (−0.739)	−0.036 (−0.678)	−0.064 (−1.031)		−0.020 (−0.386)
Q		−0.069*** (−4.298)	−0.072*** (−3.754)	−0.060*** (−2.691)		−0.085*** (−4.335)
NWC		0.023 (0.162)	−0.112 (−0.646)	0.069 (0.339)		−0.115 (−0.673)
CONS	−0.355** (−2.490)	−0.378** (−2.561)	−0.050 (−0.258)	−0.307 (−1.580)	0.435*** (5.483)	0.114 (0.779)
Year	Control	Control	Control	Control	Control	Control
Industry	Control	Control	Control	Control	Control	Control
N	7,626	7,626	5,590	4,416	4,934	4,934
R^2^	0.115	0.224	0.205	0.207	0.185	0.212

*T*-statistic values are reported in parentheses in the table. ^***^, ^**^, and ^*^ indicates that the regression results passed significance tests at the 1, 5, and 10% confidence levels, respectively.

### Regression results of mediating effects

As mentioned earlier, digitalization will have an impact on enterprises’ outbound investment through total factor productivity. Table 6 shows the regression results of mediating effect of total factor productivity & financing constraints. In column (1), the coefficient of DIG is significantly positive, verifying that digitalization has a positive impact on enterprise OFDI. In column (2), the coefficient of DIG is 0.060 and significant, indicating that digitalization can contribute to the improvement of total factor productivity. The coefficients *λ*_2_ (0.994) and *λ*_1_(0.068) of the explanatory and mediating variables on the explained variables in model (3) are also both significant at the 1% level. Besides, *β*_1_*λ*_2_ and *λ*_1_ have the same sign, indicating that total factor productivity plays a partial mediating effect between digitalization and enterprise OFDI. In reality, the impact of digital development on enterprise OFDI is multifaceted. Therefore partial mediation effect holds in reality and Hypothesis 2 is verified.

**Table 6:** Regression results of the mediating effect of total factor productivity (TFP) and financing constraint (FC).

Variables	OFDI_2	TFP	OFDI_2	OFDI_2	FC	OFDI_2
	(1)	(2)	(3)	(4)	(5)	(6)
DIG	0.128*** (5.964)	0.060*** (5.678)	0.068*** (3.242)	0.128*** (5.964)	0.019*** (8.589)	0.122*** (4.191)
TFP			0.994*** (19.696)			
FC						2.656*** (9.468)
PPE	−0.420* (−1.831)	−1.881*** (−16.886)	1.449*** (5.995)	0.420* (1.831)	−0.064*** (−2.725)	−0.452** (−1.985)
LEV	1.585*** (11.657)	0.821*** (12.601)	0.769*** (5.565)	1.585*** (11.657)	−0.081*** (−5.580)	1.730*** (12.735)
K	0.115* (1.956)	0.126*** (4.680)	−0.010 (−0.169)	0.115* (1.956)	0.061*** (10.210)	0.061 (1.035)
ROA	0.070 (0.951)	1.046*** (21.560)	−0.970*** (−10.947)	0.070 (0.951)	−0.026*** (−3.649)	0.093 (1.272)
EXPORT	0.157** (2.256)	0.074** (2.187)	0.083 (1.232)	0.157** (2.256)	0.022*** (3.460)	0.136** (1.970)
GROW	−0.032 (−0.739)	0.153*** (9.121)	−0.185*** (−4.284)	−0.032 (−0.739)	0.065*** (6.909)	−0.026 (−0.590)
Q	−0.069*** (−4.298)	−0.026*** (−4.120)	−0.043*** (−2.757)	−0.069*** (−4.298)	0.007** (2.537)	−0.076*** (−4.753)
NWC	0.023 (0.162)	0.169*** (4.630)	−0.145 (−1.036)	0.023 (0.162)	−0.033 (−0.989)	0.072 (0.505)
CONS	−0.378** (−2.561)	7.740*** (111.218)	−8.072*** (−19.403)	−0.378** (−2.561)	−3.556*** (−133.523)	8.808*** (8.976)
Year	Control	Control	Control	Control	Control	Control
Industry	Control	Control	Control	Control	Control	Control
*N*	7,626	7,626	7,626	7,626	7,626	7,626
*R* ^2^	0.224	0.695	0.271	0.224	0.184	0.236

^***^, ^**^, and ^*^ indicate that the regression results passed significance tests at the 1, 5, and 10% confidence levels, respectively.

In addition, the paper also theoretically analyzes the influence mechanism of digitalization on enterprise OFDI from the perspective of financing constraints. In order to test this influence mechanism, the mediating effect model is used and the results are shown in Table 6. The results in column (4) show that the coefficient of explanatory variable α_1_ is significantly positive. The coefficient of explanatory variable *β*_1_ (0.019) on the mediating variable in model (5) is also significantly positive at the 1% level. It demonstrates that the digitalization can significantly reduce the financing constraints of enterprises. The coefficients *λ*_2_ (2.656) and *λ*_1_ (0.122) of the explanatory and mediating variables on the explanatory variables in model (6) are also significant at the 1% level and *β*_1_*λ*_2_ and *λ*_1_have the same sign, indicating that financing constraints play a partial mediating effect between digitalization and enterprise OFDI. Therefore, Hypothesis 3 is also proved.

### Further research

#### Analysis of the impact of the G20 Summit in 2016

The G20 Summit held in China in 2016 took digital economy as the key topic of the meeting for the first time, and adopted the G20 Digital Economy Development and Cooperation Initiative, which provides a historic opportunity for the development of digital economy in the world. Since then, China has further strengthened supports for the development of the digital economy and introduced a series of policies to support the development of big data, “Internet+,” e-commerce, etc., and promoted the deep integration of the digital economy and industry by increasing investment in communication technology and network infrastructure construction, so as to foster new economic growth points. Driven by these favorable policies, enterprises have adjusted their development strategies and increased their efforts in digital transformation. Therefore, this paper selects 2016 as the critical time point to study the impact of digital development on enterprises’ OFDI before and after the change of national policies. The regression results are shown in Table 7. Column (1) is the regression result of sub-sample of 2009–2016, where the coefficient of DIG is not significant. Column (2) is the regression result of sub-sample of 2017–2020, and the coefficient of DIG is positive and significant at the 10% level. This indicates that changes in external digital policies and environment can affect the development of enterprises. Under favorable policies and environment, corporate digital transformation exerts positive influence on enterprises’ foreign direct investment.

**Table 7:** Regression results according to the “G20 Summit” time node.

Variables	OFDI_2	
	(1)	(2)
DIG	0.012 (0.475)	0.045* (1.784)
PPE	−0.687** (−2.424)	−0.509* (−1.836)
LEV	1.073*** (5.688)	1.351*** (7.689)
K	0.079 (1.002)	0.028 (0.376)
ROA	−0.247** (−2.312)	−0.250** (−2.494)
EXPORT	0.132 (1.481)	0.060 (0.709)
GROW	0.043 (0.752)	0.059 (1.112)
Q	−0.049** (−2.517)	−0.070*** (−3.613)
NWC	0.050 (0.265)	−0.123 (−0.692)
CONS	0.536*** (3.258)	0.380** (2.313)
Year	Control	Control
Industry	Control	Control
N	3,288	4,183
R^2^	0.163	0.195

^***^, ^**^, and ^*^ indicate that the regression results passed significance tests at the 1, 5, and 10% confidence levels, respectively.

#### Analysis from the perspective of different property rights

In China, enterprises with different property rights differ in terms of their business objectives, internal organizational structures, so the decision-making of OFDI will also different. Therefore, this paper divides the sample into state-owned enterprises, private enterprises and other enterprises. The regression results are shown in Table 8. Column (1) shows the results of state-owned enterprises and column (2) lists those of private enterprises. The results demonstrate that the coefficient DIG in private enterprises is significant at the 1% level, which indicates that digitalization development can have a significant impact on private enterprises’ OFDI. However, the coefficient of DIG in the state-owned enterprises is not significant. This may because state-owned enterprises are often subject to many restrictions in the process of OFDI due to the special nature of their internal organization. For private enterprises, the organizational structure is relatively flexible and they are more sensitive to the market change. At the same time, private enterprises have a strong sense of risk and can use their rich experience in competition to deal with uncertainties in overseas investment. Therefore, for private enterprises, the impact of digitalization on OFDI is more significant.

**Table 8:** Regression results for different enterprise properties.

Variables	State-owned enterprises	Private enterprises	Other enterprises
	(1)	(2)	(3)
DIG	0.050 (1.221)	0.151*** (5.456)	0.092 (1.458)
PPE	0.903** (2.156)	0.432 (1.417)	−0.053 (−0.088)
LEV	1.370*** (4.963)	1.436*** (8.371)	2.220*** (5.490)
K	0.161 (1.541)	0.096 (1.250)	0.103 (0.583)
ROA	−0.161 (−1.249)	0.175* (1.808)	−0.021 (−0.097)
EXPORT	−0.095 (−0.726)	0.377*** (4.272)	−0.636*** (−2.784)
GROW	−0.052 (−0.634)	−0.015 (−0.260)	−0.006 (−0.047)
Q	−0.046 (−1.462)	−0.054** (−2.535)	−0.155*** (−3.849)
NWC	0.284 (0.992)	−0.020 (−0.107)	−0.031 (−0.077)
CONS	0.034 (0.076)	−0.735*** (−4.017)	0.728* (1.820)
Year	Control	Control	Control
Industry	Control	Control	Control
*N*	1901	4,767	958
*R* ^2^	0.174	0.249	0.251

^***^, ^**^, and ^*^ indicate that the regression results passed significance tests at the 1, 5, and 10% confidence levels, respectively.

#### Analysis from the perspective of different industries

There are great differences in labor, capital and technology inputs in the production of products in different industries, and there are also great differences in the market competition faced by different industries, which will lead to the differences in foreign investment strategies of enterprises. Therefore, the impact of digitalization on enterprises’ outbound investment may vary from industry to industry. Consequently, we will test the regression model again according to different industries.

With reference to the method of industry segmentation by Lu and Yin (2014), based on the industry classification of China Securities Regulatory Commission (CSRC), this paper divides the manufacturing industry into 29 sub-industries, together with other 16 industries, 45 industry categories are obtained. Then it classifies industries into labor-intensive industry, capital-intensive industry and technology-intensive industry. The classification of industry categories is as follows:


(1) Percentage of fixed assets=netfixed assets/total assets

(2) R&Dexpenditure share=R&Dexpenditure/employee salary

In Equation (1), if the proportion of fixed assets is high, the company is classified into capital-intensive industry. In Equation (2), if the ratio is high, indicating that technology is important in the enterprise and the company is classified into technology-intensive industry. Enterprises that are not classified into the above two kind of industries will be classified as labor-intensive industry. Firstly, the ratios of fixed assets and R&D expenditures are calculated for 45 industries. Secondly, all industries are divided into three major categories for analysis by using Wardslinkage’s Sum of Squares of Deviations Method. The results of industry classification are shown in Table 9.

**Table 9:** Industry segmentation.

Category	Specific industries
Labor-intensive	A agriculture, forestry, animal husbandry, fishing, B mining, D electricity, heat, gas and water production and supply, E construction, F wholesale and retail trade, G transportation, storage and postal services, H accommodation and catering, P education, S comprehensive, C13 agro-food processing industry, C14 food manufacturing C15 wine, beverage and refined tea manufacturing, C17 textiles, C18 textile clothing, apparel industry, the C19 leather, fur, feathers and their products and footwear industry, C20 wood processing and wood, bamboo, rattan, palm, grass products industry, C21 furniture manufacturing.
Capital-intensive	K real estate, L rental and business services, N water, environment and public facilities management, Q health and social work, R culture, sports and entertainment, C22 paper and paper products, C23 printing and recording media reproduction industry, C24 education, industry, sports and entertainment goods manufacturing industry, C25 petroleum processing, coking and nuclear fuel processing industry, C26 chemical materials and chemical products manufacturing industry, C28 chemical fiber manufacturing industry, C29 rubber and plastic products industry, C30 non-metallic mineral products industry, C31 ferrous metal smelting and rolling processing industry, C32 non-ferrous metal smelting and rolling processing industry, C33 metal products industry.
Technology-intensive	I information transmission, software and information technology services, M scientific research and technology services, C27 pharmaceutical manufacturing, C34 general equipment manufacturing, C35 special equipment manufacturing, C36 automobile manufacturing, C37 railroad, ship, aerospace and other transportation equipment manufacturing, C38 electrical machinery and equipment manufacturing, C39 computer, communications and other electronic equipment manufacturing, C40 instrumentation manufacturing, C41 other manufacturing industries, C42 comprehensive utilization of waste resources.

The regressions results of three kinds of industries are shown in Table 10. The coefficients of DIG are significant at the 1% level in columns (1), (3) and at the 5% level in column (2). This indicates that for all types of enterprises, digitalization can significantly promote their OFDI. Bootstrap method is used to test the difference in DIG coefficients between group (1) and group (3). The sampling time was set to 1,000 times, and the coefficient difference between these two groups was significant at the 1% level of significance (0.230). The coefficient of DIG increased from 0.156 to 0.158. This means that the impact of digitalization on enterprise OFDI in technology-intensive enterprises was greater than labor-intensive enterprises. For technology intensive enterprises, they are highly sensitive to technological change and can grasp the industry trends in a timely manner. Therefore, the motive of digital transformation will be stronger, and the effect will be better. The use of digital technology will also greatly improve the ability of enterprises to operate internationally, thus promoting foreign investment. For labor-intensive enterprises, the application of digital technology is mainly reflected in the reduction of labor costs, which will make enterprises more inclined to continue to operate at home, thus the promotion of enterprises’ foreign investment is relatively weak.

**Table 10:** Regression results for different factor intensities.

Variables	Labor-intensive	Capital-intensive	Technology-intensive
	(1)	(2)	(3)
DIG	0.156*** (2.834)	0.101** (2.420)	0.158*** (4.942)
PPE	−0.003 (−0.005)	0.002 (0.005)	0.613** (2.088)
LEV	2.268*** (6.655)	1.637*** (5.472)	1.399*** (8.230)
K	0.338*** (2.824)	−0.139 (−1.057)	0.083 (1.034)
ROA	0.412*** (2.635)	−0.000 (−0.002)	−0.046 (−0.450)
EXPORT	0.625*** (4.508)	−0.533*** (−3.598)	0.197** (2.015)
GROW	0.157 (1.366)	0.001 (0.009)	−0.114** (−2.173)
Q	−0.089* (−1.719)	−0.134*** (−3.648)	−0.043** (−2.356)
NWC	0.234 (0.633)	0.002 (0.005)	−0.042 (−0.235)
CONS	−0.811** (−2.017)	0.333 (1.084)	−0.472** (−2.502)
Year	Control	Control	Control
Industry	Control	Control	Control
*N*	1,480	1855	4,291
*R* ^2^	0.234	0.239	0.244

^***^, ^**^, and ^*^ indicate that the regression results passed significance tests at the 1, 5, and 10% confidence levels, respectively.

#### Analysis from the perspective of different locations

According to the theory of regional economics, the level of regional economic development will affect the business strategy and management ideas of enterprises. Enterprises in different locations may have different decision-making behavior. In China, the eastern enterprises usually have advantages for OFDI because of convenient transportation, better economic development foundation, more advanced management concept, etc. Therefore, it divides the samples into sub samples of eastern, central and western enterprises according to the region they are located. The regression results are presented in Table 11 respectively. In column (1), the coefficient of DIG is positive and significant at the 1% level. It indicates that with the development of digital economy, the eastern enterprises can firstly enjoy the technological dividend and enhance their OFDI with the help of emerging technology. However, in comparison, there is no significant correlation between DIG and OFDI in the central and western regions. The reason may be that the economic development of the central and western regions is comparatively backward compared to the east, and the digital development and digital infrastructure are much poorer than the east region, thus the promotion effect of DIG on OFDI is greater in east region.

**Table 11:** Regression results of enterprises in the eastern, central and western regions.

Variables	Eastern	Central	Western
	(1)	(2)	(3)
DIG	0.163*** (6.772)	−0.061 (−1.002)	0.034 (0.499)
PPE	0.221 (0.857)	0.619 (0.986)	1.195* (1.673)
LEV	1.585*** (10.537)	1.573*** (3.836)	1.633*** (3.550)
K	0.020 (0.296)	−0.024 (−0.168)	0.856*** (4.728)
ROA	0.012 (0.150)	0.450** (2.331)	0.222 (0.960)
EXPORT	0.199** (2.541)	−0.092 (−0.573)	0.161 (0.643)
GROW	−0.017 (−0.340)	−0.009 (−0.082)	−0.165 (−1.244)
Q	−0.067*** (−3.824)	−0.002 (−0.036)	−0.133** (−2.391)
NWC	−0.017 (−0.108)	0.230 (0.532)	0.034 (0.074)
CONS	−0.403** (−2.485)	0.330 (0.698)	−0.705 (−1.356)
Year	Control	Control	Control
Industry	Control	Control	Control
*N*	6,200	503	923
*R* ^2^	0.225	0.291	0.251

^***^, ^**^, and ^*^ indicate that the regression results passed significance tests at the 1, 5, and 10% confidence levels, respectively.

### Robustness tests

#### Endogeneity test

The endogeneity problem may exist in this paper. On the one hand, digitalization can promote the development of enterprise OFDI. On the other hand, the development of enterprises’ overseas investment is affected by the international market, which gives birth to greater digital motivation to meet the needs of overseas consumers. At the same time, the development of overseas investment is also likely to promote the improvement of enterprises’ digital transformation capability on the basis of obtaining higher profits. In order to mitigate the impact of reverse causality, the instrumental variable method is used for further testing. Referring to Huang et al. (2019), this paper selects selects the urban post and telecommunications data in 1984 as the tool variable of enterprise digitalization. On the one hand, the development of the city’s previous communication facilities will affect the subsequent local Internet and other technology applications from the perspective of technology level and social preference, meeting the characteristics of the required relevance; On the other hand, urban post and telecommunications and other infrastructure, which mainly provide communication services, do not directly affect enterprises’ foreign investment and have the characteristics of exogeneity. In addition, urban post and telecommunications data are cross-sectional data, which cannot be directly used for regression. With reference to Zhao et al. (2020), this paper introduces the interaction term between the number of Internet users last year and the number of fixed phones per 10,000 people in each city in 1984 as a tool variable for enterprise digitalization, and conducts logarithmic processing. The regression results are shown in the Table 12. In columns (2) The coefficients of DIG are all significantly positive at the 1% level. In addition, the statistical value of Anderson canon Corr. LM significantly rejects the original hypothesis. And Cragg–Donald’s Wald *F*-statistical value is also greater than the critical value of the Stock–Yogo weak identification test at the level of 10%, rejecting the original hypothesis of weak instruments. Therefore, it can be seen that the instrumental variable selected in this paper is reasonable.

**Table 12:** Regression results of instrumental variables & replacing core explanatory variables.

Variables	OFDI_2			
	(1)	(2)	(3)	(4)
DIG	0.669*** (6.545)	0.905*** (5.238)	0.282** (2.486)	0.265** (2.325)
PPE		0.203 (0.458)		−2.214*** (−12.694)
LEV		1.710*** (10.879)		1.189*** (11.007)
K		0.752*** (10.619)		0.509*** (10.942)
ROA		−0.060 (−0.982)		0.008 (0.148)
EXPORT		0.181*** (3.000)		0.336*** (6.862)
GROW		−0.019 (−0.213)		0.065 (0.951)
Q		−0.149*** (−4.982)		−0.025 (−1.350)
NWC		0.109 (0.382)		−0.483* (−1.956)
CONS	−0.778** (−2.389)	−2.032*** (−3.562)	1.340*** (59.474)	0.830*** (9.248)
Anderson canon. Corr.	71.229	74.152		
LM statistic	[0.000]	[0.000]		
Cragg-Donald Wald	175.484	74.762		
F statistic	[16.38]	[16.38]		
Year	Control	Control	Control	Control
Industry	Control	Control	Control	Control
N	6,538	6,538	6,187	6,187
R^2^	0.193	0.250	0.001	0.062

#### Alternative core explanatory variable

This paper uses the information about digitalization disclosed in the notes to financial reports of listed companies to measure the digitalization of enterprises. Specifically, the proportion of digitalization related items in the total intangible assets at the end of the year is used as a substitute indicator for explanatory variables. Among them, the projects related to digitalization in intangible assets mainly include “software,” “management system,” “intelligent platform,” “Internet,” etc. Add up all items and calculate their proportion in the total intangible assets, and then conduct logarithmic processing to finally obtain the indicators of enterprise digitalization. The regression results are shown in Table 12. It can be seen that in columns (4) the DIG regression coefficients are significant at the 1% level, indicating the robustness of the above conclusions.

## Discussion

Many countries have encouraged enterprises to take advantage of digital economy to promote outbound investment. However, research on the impact of enterprise digital transformation on OFDI is limited. Most of existing studies focus on the development level of the national digital economy and international investment at the macro level (Fan, 2020), or the issues related to the digital transformation path of enterprises from the micro perspective (Yi et al., 2021). There is a lack of empirical tests on the impact of corporate digitalization on OFDI. This study provides an in-depth examination on this topic. Specifically, we hypothesize that corporate digitization significantly promotes OFDI (Hypothesis 1) and that this promotion effect is more pronounced among enterprises with high total factor productivity (Hypothesis 2) and low financing constraints (Hypothesis 3). Our empirical test results also confirm these hypotheses.

In further research, the article focuses on macro policy and enterprise heterogeneity perspectives to obtain more refined and valuable conclusions. From a macro perspective, national policies can guide the development of business goals. When the state enacts policies to encourage companies to implement digital transformation, the positive impact of this policy should be taken into account in the subsequent long-term or short-term goals of the company. From the perspective of enterprise heterogeneity, first of all, the digitalization of state-owned enterprises is more likely to promote enterprises’ foreign direct investment. The reason may be that, compared with other enterprises, state-owned enterprises often face the problem of lower management efficiency due to the inherent defects of their governance structure. With digital transformation, enterprises will largely improve their management efficiency due to the improvement of information efficiency, thus greatly improving their overseas competitiveness and foreign investment ability. Second, there are significant differences in economic development between the eastern, central, and western regions of China. The eastern region has an inherent advantage for OFDI due to its favorable geographical location, and relatively convenient and diversified transportation modes. In addition, due to the accumulation of past economic development, the eastern region is more sensitive to the new economic development trend, and can take the lead in internal reform based on its own conditions, which makes the enterprises in the eastern region significantly better than those in the inland region in terms of technology use. Therefore, it is more urgent for inland companies to strengthen digitalization so as to facilitate enterprises’ OFDI. Third, the focus of technological change varies from industry to industry, and the impact of digitalization is also different. For labor-intensive enterprises, the use of digital technology improves the efficiency of machinery and equipment utilization and greatly reduces labor cost. This allows companies to allocate capital more flexibly and have more funds to invest outbound. For technology-intensive industries, digital technology is much more important for their production efficiency and oversea competitiveness, so digitalization can promote enterprises’ foreign investment much more. In general, digitalization in companies of all industries can significantly promote enterprises “going global” strategies.

## Conclusion and limitations

### Conclusion and policy implications

Under the background of vigorous development of digital economy, the integration of digital economy and real economy has become the trend of future development. Therefore, China tries to give full play to its advantages in big data and application scenarios, promote the transformation and upgrading of traditional industries and the healthy and rapid development of the digital economy. Enterprises also use digital technology to further facilitate the implementation of the “go global” strategy. Based on the micro perspective of enterprises, this paper studies the impact of digitalization on enterprise OFDI from both theoretical and empirical perspectives. The study finds that digitalization can effectively promote enterprise OFDI. And it exams the impact mechanisms of corporate digitalization on OFDI from two aspects, that is, corporate digitalization can affect OFDI by improving total factor productivity and reducing financing constraints. The study also finds that both external favorable policies and the enterprises heterogeneity can have different effects on the process of digitalization affecting enterprise OFDI. The findings of this paper have the following policy implications.

1. To realize the positive role of digital development in promoting enterprise OFDI, the state should first help enterprises to promote digital transformation development effectively. The government should improve the existing market management system and approval process that may cause obstacles to digital transformation, so as to create a favorable market regulatory atmosphere for enterprise digitalization. At the same time, relevant policies should be introduced to support the enterprise digitalization, such as favorable tax policies, government subsidies and talent introduction policies, to solve the obstacles that enterprises may encounter in the process of digitalization. In addition, the government should also attach importance to the construction and improvement of digital infrastructure, build technical facilities where enterprises need them, create an integrated digital platform, which can help enterprises improve the efficiency of resource utilization and help enterprises implement “going global” strategy.
2. This paper finds that digital technology can promote the improvement of enterprises’ total factor productivity and the reduction of financing constraints, thereby promoting the development of enterprises’ outbound investment. This shows that enterprises should use digital technology to reorganize and reallocate existing resources, and improve the efficiency of resource utilization and the communication efficiency of organizational departments, which can reduce internal costs and improve productivity to get ready for enterprises’ overseas business expansion. In addition, enterprises should also pay attention to the application of digital technology to improve corporate disclosure, enhance the standardization of enterprise information disclosure, reduce the distrust of borrowers and lenders for information asymmetry, reduce the cost of investors to use corporate information, and further expand enterprise financing channels, so as to obtain sufficient funds to support the implementation of foreign investment strategies.
3. Due to the existence of enterprise heterogeneity, digitalization will exert different impacts on enterprises with different ownership nature, different industries and different locations. Therefore, state-owned and private enterprises should actively use their own data resources, and guide the implementation of the “going out” strategy of enterprises. State-owned enterprises should rely on their anti risk advantages to flexibly use enterprise resources to participate in international competition. Labor intensive enterprises should focus on the construction of digital infrastructure and cost control, while technology intensive enterprises should focus on the development of digital assets and the application of digital technology. Enterprises in the central and western regions should try to implement digitalization to break through regional restrictions and actively participate in international investment.

### Limitations and further research

This paper analyzes the impact of corporate digitalization on OFDI and its mechanism by using a sample of listed companies in China. Although it enriches previous theoretical studies to a certain extent, there are still some limitations in this paper.

First, this paper measures the corporate digitalization by crawling and compute the frequency of keywords that associate with digitalization in financial statements. Although it reflects the importance companies place on digital transformation to a certain extent, it does not include the investment amount of enterprise digitalization. In the future, we can combine the text analysis method with the indicator of digital investment to build a more reasonable index, so as to more objectively reflect the true level of enterprise digital development.

Second, the sample of this article is all listed companies, which does not contains non-listed companies and can not reflect the basic characteristics of general enterprises, especially SMEs, resulting deficiencies in the universality of research conclusions. In the future, we can expand the research sample from listed companies to non-listed companies, and appropriately extend the sample time span, which will help to obtain more general conclusions. It is also interesting to compare listed companies with other companies and explore whether the impact of DIG on OFDI in listed companies is the same as that in non-listed companies. If different, what are the reasons for such differences? These are worthy of future in-depth studies.

## Data availability statement

The original contributions presented in the study are included in the article/supplementary material, further inquiries can be directed to the corresponding author.

## Author contributions

CP and SY conceived the idea for this study. CP conducted the final revision. SY contributed to the whole write-up. HJ provided some suggestions on statistical analysis. All authors contributed to the article and approved the submitted version.

## Conflict of interest

The authors declare that the research was conducted in the absence of any commercial or financial relationships that could be construed as a potential conflict of interest.

## Publisher’s note

All claims expressed in this article are solely those of the authors and do not necessarily represent those of their affiliated organizations, or those of the publisher, the editors and the reviewers. Any product that may be evaluated in this article, or claim that may be made by its manufacturer, is not guaranteed or endorsed by the publisher.
